# Expression of LAIR-1 (CD305) on Human Blood Monocytes as a Marker of Hepatic Cirrhosis Progression

**DOI:** 10.1155/2019/2974753

**Published:** 2019-03-24

**Authors:** María Martínez-Esparza, Antonio José Ruiz-Alcaraz, Violeta Carmona-Martínez, María Dolores Fernández-Fernández, Gonzalo Antón, María Muñoz-Tornero, Miriam Lencina, Inmaculada Pagán, Jesús de la Peña, Pilar García-Peñarrubia

**Affiliations:** ^1^Departamento de Bioquímica, Biología Molecular (B) e Inmunología, Facultad de Medicina, IMIB and Regional Campus of International Excellence “Campus Mare Nostrum”, Universidad de Murcia, 30100 Murcia, Spain; ^2^Unidad de Trasplante Hepático, Servicio de Aparato Digestivo, Hospital Universitario Virgen de la Arrixaca, Murcia, Spain; ^3^Servicio de Anatomía Patológica, Hospital Universitario Virgen de la Arrixaca, IMIB, Murcia, Spain

## Abstract

**Background and Aim:**

The presumed role of the inhibitory receptor LAIR-1 (CD305) in the inflammatory response suggests that it might contribute to the pathophysiology of chronic inflammatory diseases such as liver cirrhosis. We studied the LAIR-1 expression on liver macrophages and blood monocytes related to the progression of liver cirrhosis.

**Methods:**

The expression of LAIR-1 was analyzed by immunohistochemistry, flow cytometry, and Western blot.

**Results:**

We found a decreased number of macrophages expressing LAIR-1 in cirrhotic liver that could be due to a high presence of collagen, ligand of LAIR-1, in the fibrotic tissue which could downregulate its expression or interfere with the immunostaining. The expression of LAIR-1 decreased after cell differentiation, and the total content, but not the cell surface expression, increased after activation in the HL-60 human macrophage *in vitro* model. Blood monocytes exhibited higher LAIR-1 expression levels in cirrhotic patients, which were evident even in early clinical stages in all monocyte subsets, and greater in the “intermediate” inflammatory monocyte subpopulation. The *in vitro* activation of human blood monocytes did not increase its expression on the cell surface suggesting that the *in vivo* increase of LAIR-1 must be the result of a specific combination of stimuli present in cirrhotic patients. This represents an exclusive feature of liver cirrhosis, since blood monocytes from other chronic inflammatory pathologies showed similar or lower LAIR-1 levels compared with those of healthy controls.

**Conclusions:**

These results may indicate that monocyte LAIR-1 expression is a new biomarker to early detect liver damage caused by chronic inflammation in liver cirrhosis.

## 1. Introduction

The appropriate response of the immune system against injury depends on a delicate balance between activating and inhibitory signals. A predominant immunosuppression state leads to the risk of severe infection and immunodeficiency, while insufficient inhibition may lead to a state of hyperreactivity that facilitates the development of autoimmune diseases. Inhibitory receptors of the immune response play a crucial role in maintaining this balance [1]. Traditionally, research on inhibitory receptors has focused on NK cells, B lymphocytes, and T lymphocytes, whereas studies on the expression of these receptors on phagocytic cells such as neutrophils, monocytes, and macrophages are scarce [2].

LAIR-1 (leukocyte-associated immunoglobulin-like receptor-1) is a type I transmembrane glycoprotein with an extracellular C2-type Ig-like domain and two immunoreceptor tyrosine-based inhibitory motifs (ITIM) in the cytoplasmic portion [3]. Interaction of LAIR-1 with several functional ligands, such as extracellular matrix collagens [4], the C1q complement component [5], and the surfactant protein D [6], inhibits immune cell activation or differentiation through SHP-1, SHP-2, and CSK intracellular signaling [7].

The expression of the inhibitory receptor LAIR-1 has been described in different cell types of the immune system including T and B lymphocytes, NK cells, monocytes, dendritic cells, eosinophils, mast cells, basophils, and CD34^+^ hematopoietic progenitor cells [3]. Expression of LAIR-1 has been also described in neutrophils and alveolar macrophages [8] as well as in synovial osteoclasts [9]. *In vitro* experiments have shown that cross-linking of LAIR-1 with monoclonal antibodies, or its ligands, inhibits the cytotoxic activity of NK and CD8^+^ T cells [10], the differentiation and activation of B cells induced by the B cell receptor cross-linking [11], the T cell receptor/CD3 complex signaling [12], the differentiation of peripheral blood precursors into dendritic cells (DC) [13, 14], and the production of type I interferon by CpG-activated plasmacytoid DC [15] and monocytes [5].

Altered expression of LAIR-1 levels in rheumatoid arthritis (RA) [16] and systemic lupus erythematosus (SLE) patients [17, 18] correlates with inflammation, which indicates a potential role of LAIR-1 in these chronic inflammatory diseases.

The attributed role of LAIR-1 in the inflammatory process suggests that this receptor might also contribute to the pathophysiology of other chronic inflammatory diseases such as liver cirrhosis. Liver cirrhosis is an end-stage hepatic disarrangement characterized by a progressive replacement of the functional hepatic architecture by nonfunctional fibrotic tissue rich in collagen deposition. The progression toward liver cirrhosis is caused by a dysregulation of mechanisms that govern the balance between activation/homeostasis of the immune system. Chronic liver inflammation and fibrosis are associated with dysfunctional monocyte and macrophage accumulation in the liver, which are responsible for many pathophysiological events associated with cirrhosis [19]. Blood monocytes contain three subpopulations displaying different phenotypes and functions [20] according to the expression pattern of the membrane markers CD14 and CD16 (Fc*γ*RIII), named as “classical” CD14^++^CD16^−^, “intermediate” CD14^++^CD16^+^, and “nonclassical” CD14^+^CD16^+^ monocytes [21, 22]. The CD16^+^ subpopulations are considered as proinflammatory monocytes since they are expanded in acute [23] and chronic [24–26] inflammatory processes.

The aim of this work was to study the role of LAIR-1 expression on monocytes and macrophages in the development and/or progression of liver cirrhosis. To assess this, we have analyzed LAIR-1 expression in liver biopsies and peripheral blood of cirrhotic patients. In this report, we found that LAIR-1 is expressed in hepatic macrophages, although the number of positive cells in cirrhotic liver is reduced. On the contrary, while LAIR-1 is widely expressed in peripheral blood monocytes, its expression level is higher in cirrhotic patients than in healthy controls even at early clinical stages. These results indicate that LAIR-1 could be considered as a promising biomarker for diagnosis and evaluation of hepatic cirrhosis progression in blood samples.

## 2. Material and Methods

### 2.1. Ethics Statement

The ethics committees (Comité Ético de Investigación Clínica at Hospital Clínico Universitario “Virgen de la Arrixaca” (HCUVA) and Comité de Bioética at the University of Murcia) approved the study protocol, and all patients gave informed consent for inclusion in the study.

### 2.2. Patients

Patients were admitted at the Liver Transplant Unit of the HCUVA (Murcia, Spain). Cirrhosis was diagnosed by clinical, histological, laboratory, and/or ultrasound findings. Exclusion criteria were the presence of positive blood or ascitic cultures, polymorphonuclear (PMN) leukocyte counts equal or greater than 250/*μ*l [27], signs or symptoms of systemic inflammatory response syndrome [28], upper gastrointestinal bleeding, hepatocellular carcinoma fulfilling Milan criteria [29], and/or portal vein thrombosis, previous liver transplantation, transjugular intrahepatic portosystemic shunt, age older than 80 or lower than 18 years, and refusal to participate in the study.

Blood was obtained for routine hematological, biochemical, and coagulation studies. Ascites was obtained in aseptic conditions for routine biochemical study and PMN counts. Both, blood, and ascites were injected in aerobic and anaerobic blood culture bottles, 10 ml each, at the bedside [30].

### 2.3. Immunohistochemistry

Hepatic biopsies from 23 patients with decompensated liver cirrhosis and 18 healthy controls (liver donors, *n* = 14; benign hepatic cysts, *n* = 3; and wounded, *n* = 1) were selected in the Pathology Department of HCUVA (Murcia, Spain) for immunohistochemical study.

For immunohistochemistry analysis, 3 *μ*m thick sections from liver biopsies were deparaffinized and dehydrated. The staining was performed in an automatic immunostainer (OMNIS, Dako/Agilent, California, USA) in the PT Link System (Dako) by incubating the samples in Tris/EDTA buffer pH 9 for 20 minutes at 95 ± 2°C. Next, the endogenous peroxidase was blocked by the EnVision™ FLEX Peroxidase Blocking Reagent for 5 min and a sequential staining was performed. First, sections were incubated for 20 min with monoclonal mouse anti-human CD68, clone PG-M1, ready to use (Dako/Agilent, California, USA). For development of the signal, the EnVision™ FLEX system (Dako/Agilent) was used according to the manufacturer's instructions and the reaction was visualized by HRP Magenta Chromogen. Then, sections were incubated for 20 min with monoclonal mouse anti-LAIR-1 IgG antibody 9.1C3, kindly provided by Dr. Boquan Jin (Department of Immunology, Fourth Military Medical University, China) (dilution 1 : 700). For development of the signal, the EnVision™ FLEX system (Dako/Agilent) was used with diaminobenzidine as chromogen and subsequently counterstained with hematoxylin for 10 min. All reagents except the primary antibody come from FLEX™ EnVision+ Kit (DAKO, Germany). The samples were then dehydrated and mounted for observation under the microscope. For each sample, 5 fields were randomly examined by 2 independent researchers. Images were obtained with a Leica DMD108 digital microimaging device (Leica, Microsystems Inc., Buffalo Grove, IL, USA). The number of LAIR-1- (brown) and CD68- (magenta) positive stained cells in each sample was evaluated.

### 2.4. Flow Cytometry

Blood samples and cultured cells were stained with monoclonal antibodies and analyzed by flow cytometry. Antibodies used were monoclonal mouse anti-human CD16-PE Cy5, LAIR-1-PE (BD Pharmingen, San Diego, USA), and CD14-FITC (eBioscience, San Diego, CA). The mouse IgG1-PE, IgG1-FITC, and IgG1-PE-Cy5 antibodies used as isotype controls were from BD Pharmingen. In brief, 50 *μ*l of peripheral blood was stained with 5 *μ*l of the corresponding monoclonal antibody and incubated in the dark, fixed with a fixing-lysing solution (Becton Dickinson, San José, CA), and then washed twice with PBS, resuspended in PBS, and kept at 4°C in the dark until data acquisition.

Flow cytometry analyses were performed on three-color fluorescence EPICS XL or FC500 (Beckman Coulter) using CYTOMICS RXP Analysis Software or version 2.5.1 of Flowing Software. 30,000-200,000 gated events were acquired and analyzed. Leukocytes were gated based on FSC *vs.* SSC (forward *vs.* side scatter) on a lineal scale. Then, mononuclear myeloid cells were gated on the base of both CD14^+^ cells and morphology. This gate was used to analyze LAIR-1 expression. CD14^+^ and CD16^+^ cells were measured as a percentage of the total number of mononuclear myeloid cells. The median fluorescence intensity (MFI) of each marker represents the density of the corresponding membrane receptor expressed per cell. Isotype control and fluorescence minus one (FMO) assays were carried out in order to establish fluorescence control limits and to ensure no spread of fluorescence between channels, respectively.

### 2.5. Cell Lines and Culture Conditions

HL-60 cells were cultured in suspension in RPMI 1640 (PAA, Pasching, Austria) with L-glutamine (PAA, Pasching, Austria) supplemented with 10% fetal calf serum and 1% penicillin-streptomycin (MCC, complete culture medium) at 37°C and 5% CO_2_. Cells were cultured at 2 × 10^6^ cells/well in 6-well plates and differentiated into macrophage-like cells with 10 ng/ml phorbol 12-myristate-14-acetate (PMA; Sigma-Aldrich Co., USA) for 24 h. Subsequently, the medium was replaced by fresh MCC for 24 hours.

Blood obtained from healthy individuals was diluted with sterile PBS and placed on Ficoll (Axis Shield Poc AS, Oslo, Norway) in Falcon tubes to perform the separation of the lymphomonocytic portion by centrifugation. The cells obtained were then washed and counted. The proportion of monocytes in the sample was determined by flow cytometry as described above. The cells were seeded at a concentration of 2 × 10^6^ monocytes/well in 6-well plates for Western blotting assays and 2 × 10^5^ monocytes/well in 24-well plates for flow cytometry analysis. After overnight incubation at 37°C and 5% CO_2_, cells were washed with MCC to remove nonadherent cells, including lymphocytes. The purity of M-DM in the cell cultures was higher than 95%.

The cell cultures were stimulated with 0.1 *μ*g/ml LPS (*E. coli* serotype 0111.B4, Sigma-Aldrich Co., USA) or heat-killed *C. albicans* (SC5314 strain) at ratio 1 : 5 cell : yeast, for 24 hours.

Cells were detached from the culture wells with Accutase or TrypLE Express 12601 (GIBCO, Invitrogen, Paisley, UK) for flow cytometry analysis or directly solubilized on the wells for Western blotting.

### 2.6. Preparation of Cell Extracts and Western Blotting

After incubation with each stimulus, the cultured cells were washed with cold PBS. The cells were then solubilized using a commercial lysis buffer (Cell Signaling Technology®, Boston, USA) and 1 mM of the protease inhibitor PMSF (phenylmethylsulfonyl fluoride) (Sigma-Aldrich Co., Saint Louis, MO, USA). The rupture of the cells was facilitated using a cell scraper (Sarstedt, Nümbrecht, Germany). The cell extract obtained was centrifuged at 4°C for 15 min at 13000 rpm, and the precipitate was discarded. The quantification of protein in the cell extract was performed by Bradford assay.

The samples were mixed with a loading buffer, heated for 5 min at 95°C, separated on a 12% acrylamide gel (15 *μ*g protein/lane) with Mini-PROTEAN II (Bio-Rad Laboratories, California, USA), and transferred by a wet system Millipore™ membranes of PVDF (0.45 *μ*m, Millipore Corporation, Bedford, MA, USA) in a transfer tray Mini Trans-Blot Bio-Rad. After blocking for 1 h at room temperature, in 3% BSA-PBS-T (0.05% Tween 20), the membrane was incubated with the monoclonal mouse IgG (clone 9.1C3) for human LAIR-1 detection (1 : 10000 dilution) or with the rabbit polyclonal IgG for human *β*-actin (1 : 5000 dilution) (Sigma-Aldrich Co., St. Louis, MO, USA), in 2% BSA-PBS-T at 4°C overnight. Then, samples were incubated with specific secondary anti-rabbit or anti-mouse antibody conjugated with horseradish peroxidase (HRP) (Santa Cruz Biotechnology Inc., Santa Cruz, California, USA) at 1 : 20000. The activity of membrane-bound peroxidase was detected by using an enhanced chemiluminescent detection method (Enhanced Chemiluminescence System©, Amersham Pharmacia Biotech, Piscataway, NJ, USA). The obtained protein bands were quantified by densitometry using the Scion Image software (Scion Inc., USA). The membranes were first blotted for LAIR-1 and then stripped for 30 minutes at room temperature with a stripping solution (*β*-mercaptoethanol 0.1 M, Tris-HCl 0.62 mM pH 6.8, and SDS 2%), washed with PBS-T, and then reprobed for *β*-actin. The results of LAIR-1 expression are referred to as the expression of the loading control, *β*-actin.

### 2.7. Statistical Analysis

The results are reported as follows: (i) mean ± standard error of the mean (SEM), represented graphically as histograms or dot plots, or (ii) median and interquartile range (IQR).

*U* Mann-Whitney test and paired Student's *t*-test were applied to determine the existence of significant statistic differences. All reported *P* values are two-sided, and *P* values lower than 0.05 were considered to indicate statistical significance.

For graphical representation of the results and statistical treatment of data, the GraphPad Prism version 5.00 for Windows was used (GraphPad Software, San Diego California USA).

Reporting of the study conforms to STROBE and EQUATOR guidelines [31].

## 3. Results

### 3.1. Clinical Characteristics of Patients

We studied a consecutive series of 44 patients with liver cirrhosis, from which a group of 39 patients fulfilling all the inclusion criteria were selected for this investigation. Causes of exclusion of the five remaining patients were hepatocellular carcinoma (*n* = 2), human immunodeficiency virus infection (*n* = 1), and culture-positive ascites (*n* = 2). Patients with compensated cirrhosis (*n* = 7) do not have symptoms related to their cirrhosis, but may have asymptomatic esophageal or gastric varices. Patients with decompensated cirrhosis (*n* = 32) have symptomatic complications related to cirrhosis, including those related to hepatic insufficiency (jaundice) and those related to portal hypertension (ascites, variceal hemorrhage, or hepatic encephalopathy). The disease etiology was ALC-C (*n* = 18), HCV-C (*n* = 11), cryptogenic cirrhosis (CRYP-C) (*n* = 8), autoimmune cirrhosis (*n* = 1), and Wilson disease (*n* = 1). None of the patients died during hospitalization or developed spontaneous bacterial peritonitis. The study was performed in parallel with healthy controls (*n* = 38). The clinical and analytical characteristics of the subjects included in this study are shown in Table 1.

### 3.2. LAIR-1 Protein Is Expressed in Human Liver Macrophages but Not in Hepatocytes

**Figure 1:**
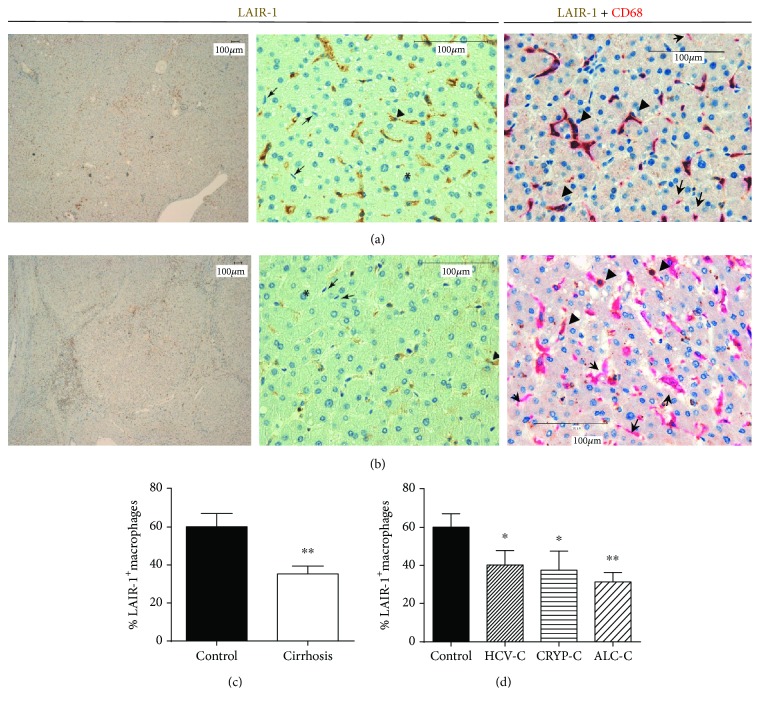
LAIR-1 expression in cirrhotic human liver. LAIR-1 expression was analyzed by immunohistochemistry in hepatic biopsies from controls (*n* = 18) (a) and cirrhotic patients (*n* = 22) (b). Images were obtained using a Leica DM108 with a magnification of 4x (left panels) and 40x (middle and right panels). The asterisks show hepatocytes, the arrowheads show macrophages with positive staining, and the arrows point at macrophages with negative staining for LAIR-1. The number of macrophages (CD68 positive staining in magenta) expressing LAIR-1 (brown) was quantified and compared between control and cirrhosis (c) or between healthy controls and cirrhotic patients attending to their etiology (d). Data represented in histograms are the mean and SEM. Two-sided Mann-Whitney *U* test: ^∗^*P* < 0.05 and ^∗∗^*P* < 0.01, between cirrhotic patients and healthy controls.

LAIR-1 expression was analyzed by immunohistochemistry on liver biopsies from 18 healthy controls and 22 patients diagnosed with liver cirrhosis (Figure 1). Representative images of liver sections, taken at low magnification, reveal the loss of hepatic tissue structures and the presence of nodules surrounded by fibrotic septa in patients with liver cirrhosis (Figure 1(b)) compared with healthy controls (Figure 1(a)). At higher magnification, the images reflect the loss of the elongated nature of the sinusoids in the case of cirrhotic livers compared with healthy controls.

Hepatocytes had a negative reaction with the antibody 9.1C3 in all cases, while macrophages displayed a positive LAIR-1 staining in both the cytoplasm and membrane, with a diffuse granular staining pattern (Figures 1(a) and 1(b)).

The number of macrophages (identified by morphology and CD68 staining) expressing LAIR-1 was quantified and represented as histograms (Figure 1(c)). Our results showed that liver biopsies from cirrhotic patients presented a lower number of LAIR-1-positive macrophages than those of healthy controls did (*P* = 0.0036) (Figure 1(c)). There were not significant differences in the number of LAIR-1+ macrophages related to the etiology of liver cirrhosis (Figure 1(d)).

### 3.3. LAIR-1 Expression Levels on Blood Monocytes from Cirrhotic Patients Are Higher than Those of Healthy Controls

**Figure 2:**
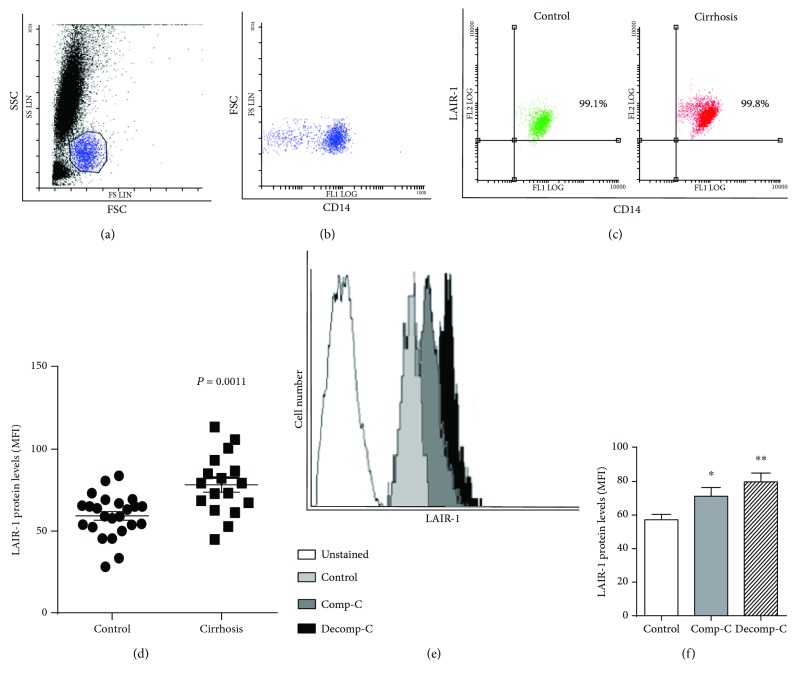
LAIR-1 expression in blood monocytes. LAIR-1 expression was analyzed by flow cytometry in peripheral blood of cirrhotic patients (*n* = 17) or healthy controls (*n* = 20). Leukocytes were gated based on forward *vs.* side scatter (a). Monocytes were gated on the base of both CD14^+^ cells and morphology (b). Representative dot-plot of monocytes stained with CD14 and LAIR-1 antibodies in healthy controls or cirrhotic patients (c). The cell surface LAIR-1 protein expression was quantified measuring the MFI (median fluorescence intensity) after cell staining and compared between control and cirrhosis (horizontal lines indicate the mean and SEM) (d). Representative histograms of monocytes unstained and stained with LAIR-1 antibody in healthy controls and compensated (Comp-C) or decompensated (Decomp-C) cirrhotic patients are shown (e). The cell surface LAIR-1 protein expression was compared between healthy controls and cirrhotic patients attending to their clinical progression, compensated (Comp-C) or decompensated (Decomp-C) cirrhosis, and represented in histograms (bars represent the mean and SEM) (f). Two-sided Mann-Whitney *U* test: ^∗^*P* < 0.05 and ^∗∗^*P* < 0.01, between cirrhotic patients and healthy controls.

Once we detected alterations in LAIR-1 expression in liver macrophages, we analyzed its expression on blood monocytes, as they are recruited and differentiated into macrophages in inflamed tissues. The number of cells expressing LAIR-1 and the amount of this receptor expressed per cell (MFI, median fluorescence intensity) were analyzed by flow cytometry in blood monocytes from 20 healthy controls and 17 patients diagnosed with liver cirrhosis (Figure 2). The results revealed that more than 99% of blood monocytes expressed LAIR-1 on the cell surface, both in healthy controls and in cirrhotic patients (Figure 2(c)). However, the amount of LAIR-1 expressed per cell (MFI) was statistically higher in blood monocytes from patients with cirrhosis compared to healthy donors (*P* = 0.0011) (Figure 2(d)). These differences were maintained when the patients were grouped attending to the stage of clinical progression or classification, i.e., the LAIR-1 expression increased in blood monocytes even in early clinical stages of cirrhosis (*P* = 0.0459 compensated cirrhosis, and *P* = 0.0017 decompensated cirrhosis) (Figures 2(e) and 2(f)).

### 3.4. LAIR-1 Expression Increased in All Subsets of Blood Monocytes in Cirrhotic Patients

**Figure 3:**
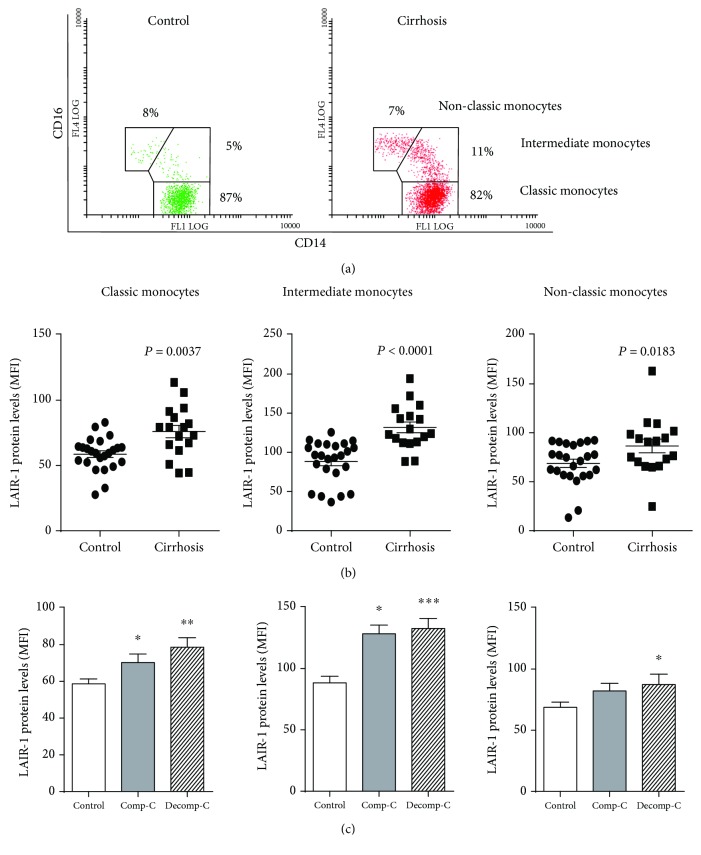
LAIR-1 expression in blood monocyte subsets. LAIR-1 expression in peripheral blood of cirrhotic patients (*n* = 17) or healthy controls (*n* = 20) was analyzed by flow cytometry. The population of monocytes was gated on the base of morphology and CD14^+^ expression. (a) Representative dot-plot of CD14 and CD16 expression pattern on blood monocytes and gates selecting the three different CD14/CD16 subsets CD14^++^CD16^−^ (classic), CD14^++^CD16^+^ (intermediate), and CD14^low^CD16^+^ (nonclassical) monocytes, in healthy controls and cirrhotic patients. Percentages refer to the mean ratio of cell subpopulations gated on the total population of CD14+ cells. (b) The LAIR-1 expression was compared between control and cirrhosis within each monocyte CD14/CD16 subset (horizontal lines indicate the mean and SEM) (c) or between healthy controls and cirrhotic patients attending to their clinical progression, compensated (Comp-C) or decompensated (Decomp-C) cirrhosis (bars represent the mean and SEM). Two-sided Mann-Whitney *U* test: ^∗^*P* < 0.05, ^∗∗^*P* < 0.01, and ^∗∗∗^*P* < 0.001, between cirrhotic patients and healthy controls.

We further studied LAIR-1 expression on the three blood monocyte subpopulations in terms of the CD14/CD16 expression. The results (Figure 3(a)) showed that the percentage of intermediate monocytes (CD14^++^CD16^+^) was increased in cirrhotic patients (11 ± 1.3% compared to 4 ± 0.4% in the blood of healthy donors, *P* < 0.0001). This increment was accompanied by a significant decrease in the CD14++CD16− classical subset (82 ± 2.0% compared to 88 ± 0.4% in healthy donors, *P* = 0.049), while no difference was observed in the case of the CD14+CD16++ nonclassical subset (8 ± 0.8%*vs*. 7 ± 0.8%, *P* = 0.21, in the blood of healthy and cirrhotic patients, respectively). All monocyte subpopulations expressed LAIR-1 in both healthy controls and cirrhotic patients, although the highest expression level was achieved within the intermediate subset (Figure 3(b)). Furthermore, the expression level of LAIR-1 (MFI) was higher in all monocyte subsets from cirrhotic patients (*P* = 0.0037 classical, *P* < 0.0001 intermediate, and *P* = 0.0183 nonclassical monocytes). Differences of LAIR-1 expression between healthy controls and cirrhotic patients were more accentuated within the intermediate monocyte subpopulation (Figure 3(b)).

Differences were also maintained when patients were grouped attending to their clinical progression. Hence, expression of LAIR-1 was increased in all blood monocyte subsets in advanced stages of cirrhosis (*P* = 0.0005 classical, *P* ≤ 0.0001 intermediate, and *P* = 0.0347 nonclassical monocytes) and even at early clinical stages of the disease for classical (*P* = 0.0493) and intermediate monocytes (*P* = 0.0175) (Figure 3(c)).

### 3.5. The Increase in LAIR-1 Protein Expression Induced by Activation Is Not Reflected on the Cell Surface in HL-60 Human Macrophage In Vitro Model

**Figure 4:**
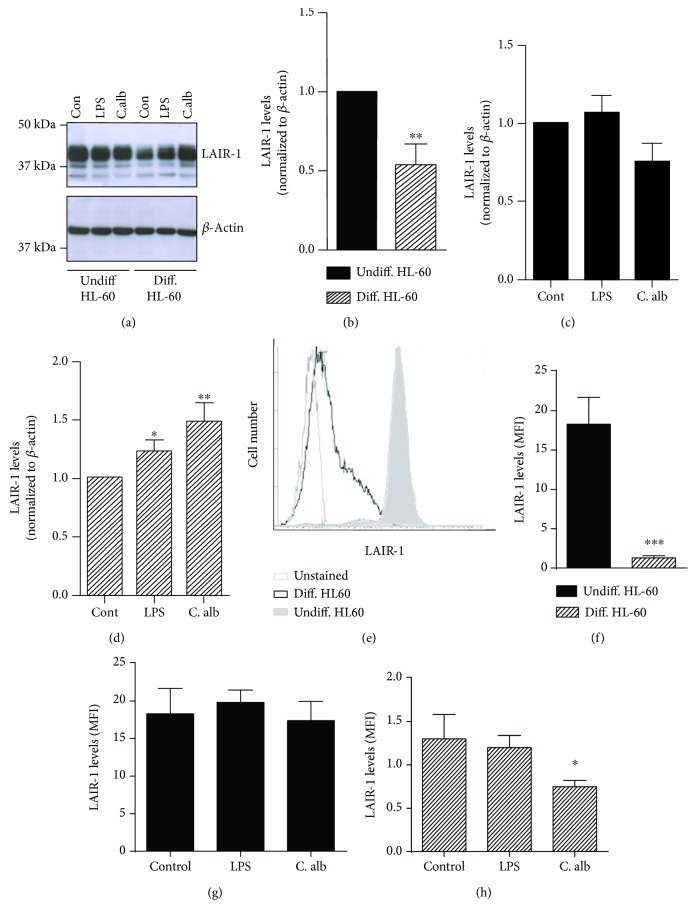
Effect of differentiation and activation on LAIR-1 expression in a macrophage-like *in vitro* model. The expression of LAIR-1 was analyzed by Western blot in total protein extracts from the HL-60 cell line undifferentiated or differentiated into macrophages with PMA treatment and unstimulated (Con, control) or stimulated with LPS or *C. albicans* 1 : 5 cell : yeast ratio. A representative Western blot is shown (a). The bands were quantified by densitometry and normalized to *β*-actin. The normalized value was referred to the undifferentiated control (normalized to 1) (b), to its own untreated control in undifferentiated HLA-60 cells (c) and in differentiated HL-60 cells (d). LAIR-1 expression was analyzed by flow cytometry. Representative histogram of HL-60 cells unstained and stained with anti-LAIR-1 antibody in undifferentiated and differentiated cells is shown (e). The cell surface LAIR-1 protein expression was quantified by measuring the MFI (median fluorescence intensity) after cell staining, and the effect of differentiation process (f) or the activation with LPS or *C. albicans* was analyzed in undifferentiated (g) or differentiated HL-60 cells (h). Data represented in histograms are the mean and SEM. Student's *t*-test: ^∗^*P* < 0.05, ^∗∗^*P* < 0.01, and ^∗∗∗^*P* < 0.001.

In order to understand the differences found in the expression of LAIR-1 in hepatic macrophages and blood monocytes of cirrhotic patients, we next studied the effect of cell differentiation and activation in the expression of LAIR-1 using the human promyelocytic cell line HL-60 as a human macrophage *in vitro* model. For this purpose, the cell line was differentiated with PMA and stimulated with LPS or *C. albicans*. The LAIR-1 total protein expression was analyzed by Western blotting, and results are shown in Figure 4. Results showed that the basal expression of LAIR-1 is reduced after differentiation (Figures 4(a) and 4(b)). Stimulation with LPS and *C. albicans* increased the LAIR-1 protein expression only in differentiated HL-60 cells (Figure 4(d)), but it did not affect the expression level in undifferentiated cells (Figure 4(c)).

Further analyses of LAIR-1 cell surface expression by flow cytometry revealed that this molecule is drastically downregulated after differentiation (Figures 4(e) and 4(f)), with the differences on expression levels being more evident on the cell surface than in the whole protein cell content (Figure 4(b)). Stimulation with LPS and *C. albicans* did not affect the LAIR-1 surface expression levels in undifferentiated cells (Figure 4(g)) which is consistent with the results found for the total LAIR-1 cell content (Figure 4(c)). Nevertheless, stimulation with LPS did not vary significantly the LAIR-1 expression, while *C. albicans* decreased the LAIR-1 protein expression on the cell surface after 24 h of treatment (Figure 4(h)), which is in contrast with the increase in total LAIR-1 protein expression registered in differentiated HL-60 cells (Figure 4(d)). These results could be explained by an accumulation of LAIR-1 protein inside the cell in response to activation, due either to inner cell retention of the protein or to its cleavage from the cell membrane.

### 3.6. Cell Surface Expression of LAIR-1 Did Not Increase after In Vitro Activation in Healthy Human Monocytes

**Figure 5:**
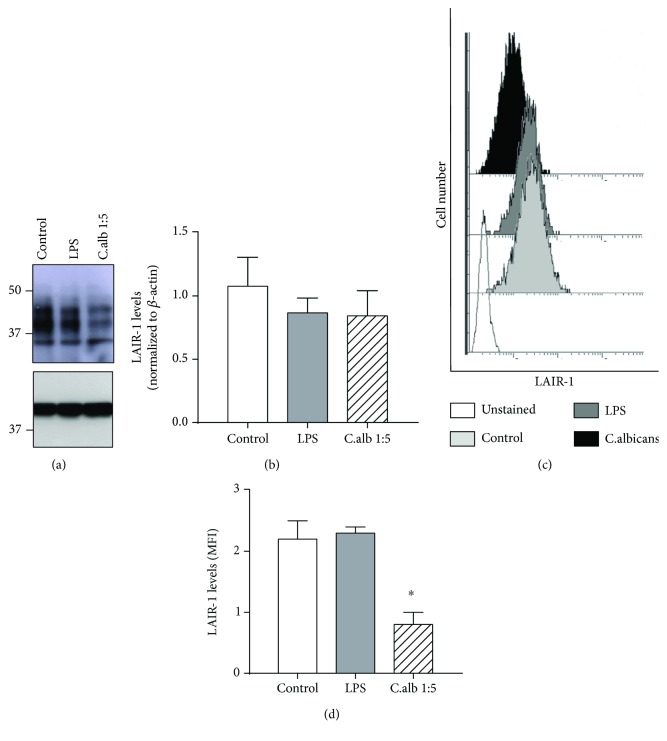
Effect of activation on LAIR-1 expression in healthy monocytes *in vitro*. The expression of LAIR-1 was analyzed by Western blotting in total protein extracts from isolated human healthy monocytes (*N* = 3) unstimulated (control) or stimulated with LPS or *C. albicans* 1 : 5 cell : yeast ratio. A representative Western blot is shown (a). The bands were quantified by densitometry and normalized to *β*-actin. (b). LAIR-1 expression was analyzed by flow cytometry after LAIR-1 and CD14 staining. Representative histograms of monocytes unstained and stained with LAIR-1 antibody in isolated monocytes are shown (c). The cell surface LAIR-1 protein expression was quantified by measuring the MFI (median fluorescence intensity) after cell staining, and the effect of the activation with LPS or *C. albicans* was analyzed (d). Data represented in histograms are the mean and SEM. Student's *t*-test: ^∗^*P* < 0.05.

Next, analysis of LAIR-1 protein expression level in healthy human monocytes was also carried out (Figure 5). For this, Western blot assays in total protein extracts were performed, and the results shown in Figures 5(a) and 5(b) revealed a different expression pattern of LAIR-1 from that found in HL-60 cells (Figure 4(a)). The different expression pattern between cell types could reflect different RNA alternative splicing and posttranslational modifications. Western blot assays were performed with a monoclonal antibody recognizing an epitope located at the N-terminus of LAIR-1 protein, which is common to the described LAIR-1 isoforms, and the presence of O-linked glycosylation sites in some of them may account for size differences on SDS-PAGE between LAIR isoforms described before [10].

The stimulation of healthy monocytes with LPS or *C. albicans* did not vary significantly the LAIR-1 total protein expression after 24 h of treatment (Figures 5(a) and 5(b)), while flow cytometry analysis revealed similar LAIR-1 surface expression levels after LPS stimulation and a decrease in response to *C. albicans* (Figures 5(c) and 5(d)).

## 4. Discussion

Monocytes and macrophages play a crucial role in the pathogenesis of chronic liver diseases [32, 33]. The progression toward liver cirrhosis is caused by a dysregulation of mechanisms that govern the balance between activation/homeostasis of the immune system [19]. Inhibitory receptors of the immune response play a key role in maintaining this balance; however, studies on cell expression of these receptors on macrophages are scarce [2].

In this work, we have studied the LAIR-1 inhibitory receptor expression in blood monocytes and liver macrophages from cirrhotic patients, trying to uncover its possible involvement in the immune-regulation of this chronic inflammatory disease. The results revealed the lack of LAIR-1 expression in hepatocytes, which is consistent with data available in human protein database [34]. Moreover, although LAIR-1 was expressed in liver macrophages, the frequency of cells expressing this receptor varied and was lower in cirrhotic patients compared to liver from healthy controls. Decreased levels of LAIR-1 in other cell types have been reported, like B lymphocytes [18], plasmacytoid DC [15], and blood monocytes [35] in SLE patients, and T lymphocytes in RA patients [9], compared with healthy controls. However, and in contrast with our data, Zhang and collaborators described, by immunohistochemistry, a higher expression of LAIR-1 in macrophages from inflamed tissue (synovial tissue samples) of RA patients compared with osteoarthritis patients (who presented joint degradation with no detectable or mild inflammation) and lower expression in healthy controls [9]. Their data are in concordance with our results obtained with the cell line HL-60, a broadly used human macrophage *in vitro* model [36, 37], which revealed that differentiation reduces the expression of LAIR-1 as reported for human neutrophils [7] and dendritic cells [13, 14], but activating stimuli such as LPS or *C. albicans* increased the total expression of LAIR-1 in differentiated or “macrophage-like” HL-60 cells. Nevertheless, flow cytometry analysis revealed that the total LAIR-1 increase did not go paralleled to the cell surface level after *in vitro* activation in macrophage-like differentiated HL-60 cells. Differences in expression of LAIR-1 in human tissue macrophages between cirrhosis and RA, both chronic systemic inflammatory diseases, could be caused by their opposite effects on tissue collagen, the known ligand for LAIR-1 receptor, among others. Thus, while cirrhotic progression is characterized by fiber formation and collagen deposition in the liver, progression of RA causes collagen destruction in the joints of the patients. Therefore, the reduction of macrophages expressing LAIR-1 protein in the liver of cirrhotic patients could be related to a downregulation of LAIR-1 after collagen binding, although interferences with antibody recognition due to collagen binding may not be discarded. In this regard, it has been described that the signaling mediated by engagement of the LAIR-1 receptor can inhibit macrophage (osteoclasts) differentiation [9] and regulate their polarization, i.e., the differentiation towards proinflammatory M1 macrophages or anti-inflammatory M2 macrophages, depending on the molecules involved [35], which can contribute to the immune dysfunction described during progression of liver cirrhosis [19].

On the other hand, we found that LAIR-1 is broadly expressed in almost all blood monocytes as previously described [9], and its density of expression was increased in cirrhotic patients compared with healthy controls, which would argue in favor of a predominant activated state of these cells in the blood of cirrhotic patients. In this regard, we have previously described the preactivated “alert state” of ascites macrophages from decompensated cirrhotic patients [38]. However, we found that the expression of LAIR-1 on the cell surface did not increase in response to *in vitro* activation with LPS and *C. albicans* in human healthy blood monocytes, which is in contrast to the results published by Ouyang et al. [39] where *in vitro* stimulation of human PBMC cells with PHA or PMA increased LAIR-1 on the cell surface. These results suggest that the LAIR-1 expression is regulated not only depending on the activation state of monocytes but also on the type of activating stimuli, and so, the *in vivo* increase in LAIR-1 expression detected in cirrhotic patients must be due to a complex combination of proinflammatory stimuli specific for this pathology. The differences in blood monocyte LAIR-1 expression between cirrhotic patients and healthy controls were evident even at early stages of cirrhosis progression. Moreover, in agreement with previous reported data, we found that the percentage of intermediate monocyte subset was not only increased in cirrhotic patients [26] but also displayed the highest LAIR-1 density expression levels and the stronger differences compared with healthy controls. This high expression would indicate an activated and “more sensitive to inhibition” status in this subpopulation. Up to date, the increased expression of LAIR-1 in blood monocytes represents an exclusive event of liver cirrhosis, unlike other chronic inflammatory pathologies such as RA [9] or SLE [35], in which blood monocytes showed similar or lower levels, respectively, compared with healthy controls.

Consistent with our results in cirrhotic patients, Zhang and collaborators described a different modulation of LAIR-1 expression in peripheral blood monocytes compared with tissue macrophages in RA patients with respect to healthy controls [9]. They showed a higher expression of LAIR-1 in synovial macrophages in RA patients compared with healthy controls [9]. However, the frequency and expression levels of LAIR-1 in peripheral blood monocytes of healthy controls compared with those of RA by flow cytometry were similar. These results point out to a different regulation of LAIR-1 expression, depending not only on the inflammatory status but also on the cell type, the differentiation state, and the pathological scenario.

These findings open a vast avenue to further investigate the functional role of LAIR-1 in the physiopathology of liver cirrhosis.

In summary, these results show that expression of LAIR-1 decreased after cell differentiation, and the total content, but not the cell surface expression, increased after activation in a human macrophage *in vitro* model. We found a decreased number of macrophages expressing LAIR-1 in cirrhotic liver that could be due to a high presence of collagen, ligand of LAIR-1, in the fibrotic tissue, which could downregulate its expression, and affect the macrophage polarization and progression of the immune dysfunction in cirrhotic liver. Blood monocytes exhibit higher LAIR-1 expression levels in cirrhotic patients, which are evident even in early clinical stages, in all monocyte subsets, and greater in the intermediate subpopulation. The *in vitro* activation of human blood monocytes with LPS and *C. albicans* did not increase its expression on the cell surface, suggesting that the *in vivo* increase of LAIR-1 in cirrhotic patients must be the result of a complex combination of proinflammatory stimuli specific for this pathology. The increased LAIR expression in blood monocytes represents an exclusive feature of liver cirrhosis, since blood monocytes from other chronic inflammatory pathologies such as RA or SLE showed similar or lower LAIR-1 levels compared with those of healthy controls.

These results suggest that monocyte LAIR-1 expression could be proposed as a new biomarker to early detect liver damage caused by chronic inflammation in liver cirrhosis.

## Figures and Tables

**Table 1:** Clinical characteristics of subjects included in the study.

Variable	Cirrhotic patients (*n* = 39)	Healthy controls (*n* = 38)
Age (years)	59 (12)	48 (20)
Sex male (*n*, %)	24, 61.5%	19, 50%
Blood WBC (number/mm^3^)	5.2 (4)	4 (3.9)
Blood monocytes (%)	6.9 (3.8)	5.9 (2.5)
Etiology of cirrhosis		
ALC (*n*, %)	18, 46.2%	—
HCV (%)	11, 28.2%	—
CRYPT (%)	8, 20.5%	—
Others (%)	2, 5.1%	—
Decompensation (*n*, %)	32, 82.0%	—
Previous ascites (*n*, %)	23,59.0%	—
Child-Pugh (score)	9 (3)	—
Child-Pugh B/C (%)	74.4/18	—
Meld (score)	15.4 (5.7)	—

Continuous variables are expressed as median (IQR) and categorical variables as percentages. Other causes of cirrhosis included Wilson disease and autoimmune cirrhosis.

## Data Availability

The data used to support the findings of this study are included within the article.

## Abbreviations

LAIR-1: Leukocyte-associated immunoglobulin-like receptor-1
ITIM: Immunoreceptor tyrosine-based inhibitory motifs
DC: Dendritic cell
M-DM: Monocyte-derived macrophages
RA: Rheumatoid arthritis
SLE: Systemic lupus erythematosus
HCV: Hepatitis C virus
HCV-C: HCV-induced cirrhosis
ALC-C: Alcohol-induced cirrhosis
PMN: Polymorphonuclear leukocytes
CRYP-C: Cryptogenic liver cirrhosis.
